# Anesthesia Techniques and Long-Term Oncological Outcomes

**DOI:** 10.3389/fonc.2021.788918

**Published:** 2021-12-08

**Authors:** Maria F. Ramirez, Juan P. Cata

**Affiliations:** ^1^ Department of Anesthesiology and Perioperative Medicine, The University of Texas MD Anderson Cancer Center, Houston, TX, United States; ^2^ Anesthesiology and Surgical Oncology Research Group, Houston, TX, United States

**Keywords:** anesthesia, analgesia, cancer recurrence, metastasis, general anesthesia (GA), regional anesthesia - palliative care - cancer pain, opioids, total intravenous anaesthesia (TIVA)

## Abstract

Despite advances in cancer treatments, surgery remains one of the most important therapies for solid tumors. Unfortunately, surgery promotes angiogenesis, shedding of cancer cells into the circulation and suppresses anti-tumor immunity. Together this increases the risk of tumor metastasis, accelerated growth of pre-existing micro-metastasis and cancer recurrence. It was theorized that regional anesthesia could influence long-term outcomes after cancer surgery, however new clinical evidence demonstrates that the anesthesia technique has little influence in oncologic outcomes. Several randomized controlled trials are in progress and may provide a better understanding on how volatile and intravenous hypnotics impact cancer progression. The purpose of this review is to summarize the effect of the anesthesia techniques on the immune system and tumor microenvironment (TME) as well as to summarize the clinical evidence of anesthesia techniques on cancer outcomes.

## Introduction

Cancer is a major global health concern since it is the second cause of death after cardiovascular disease (1). According to the World Health Organization, an estimated 19.3 million new cancer cases were recorded in 2020 with almost 10 million cancer deaths worldwide (2). In addition, given the unprecedented effects of the COVID-19 pandemic on the health care system, many patients received a delayed diagnosis and treatment (including surgery) which will significantly impact their cancer prognosis. The American Cancer Society estimates an additional 25.7 million new cancer cases worldwide and 16.3 million cancer deaths by 2040 (3). This upward trend may be secondary to earlier cancer diagnosis and improvement in prevention and treatments.

Cancer treatment may involve a combination of chemotherapy, radiotherapy, immunotherapy and surgery. The latter is also used to provide diagnosis and palliative therapy for solid tumors. While surgical excision continues to be the gold standard treatment for cancer, accumulative evidence (mostly from preclinical studies) has suggested that surgery itself and multiple perioperative events (i.e., blood transfusion, analgesics and anesthetics) might accelerate the progression of minimal residual disease, formation of new metastatic foci and cancer recurrence (4). In this review, we will focus on key mechanisms that allow surgery to provide suitable conditions for shedding, implantation and subsequently proliferation or circulating tumor cells (CTCs). Additionally, we will provide a comprehensive review of the pre-clinical data on the effect of anesthesia technique (total intravenous anesthesia [TIVA] versus volatile anesthesia) and analgesia (regional versus opioid based techniques) on cancer cells, the TME and immunosurveillance. Lastly, we will summarize the clinical data regarding the effects of the anesthesia techniques on cancer outcomes including survival.

## The Role of Surgery in Cancer Progression

### Surgery Triggers Inflammation Followed by Immunosuppression

**Figure 1:**
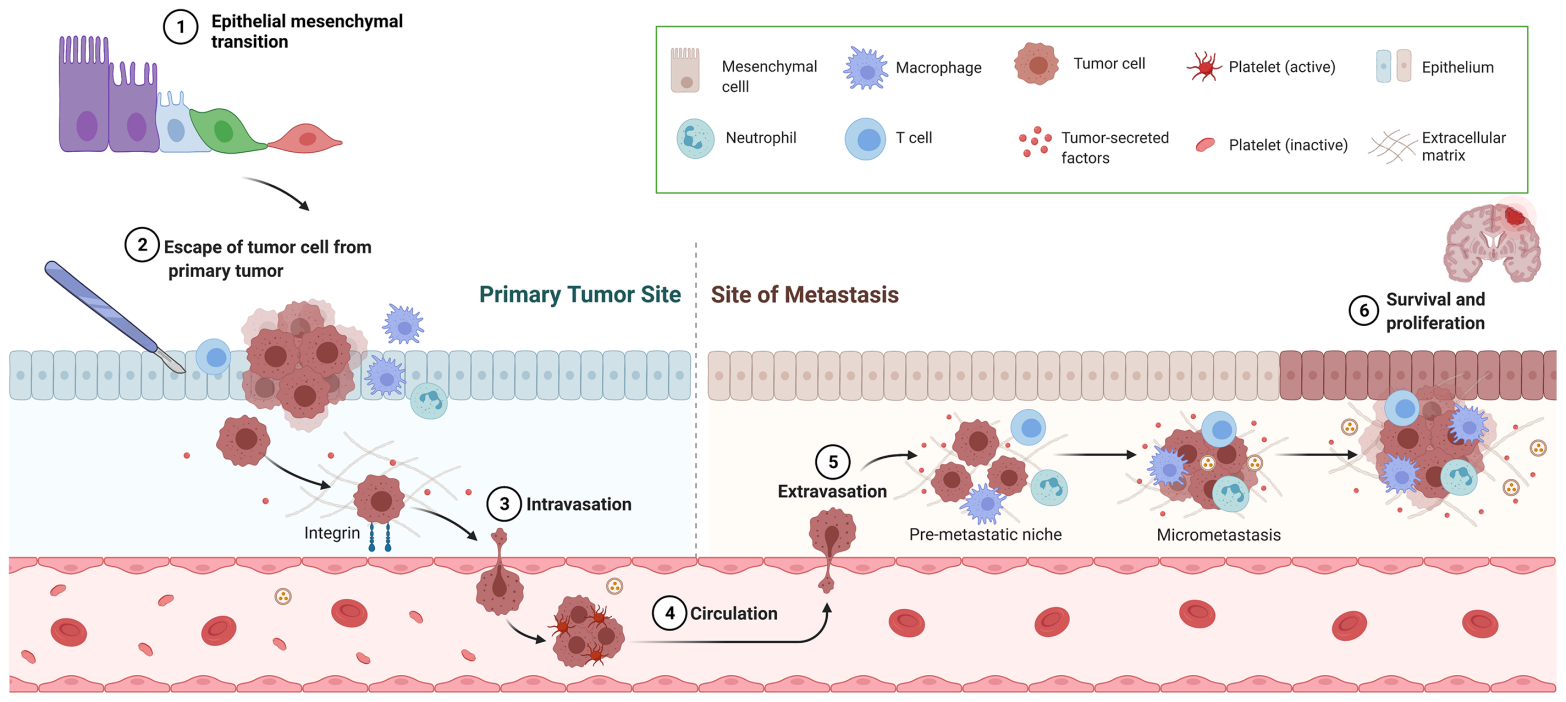
Overview of metastatic cascade. This figure represents the necessary steps for successful metastasis including epithelial-mesenchymal transition, escape of tumor cell from primary tumor, intravasation, circulation, extravasation and survival and proliferation.

**Figure 2:**
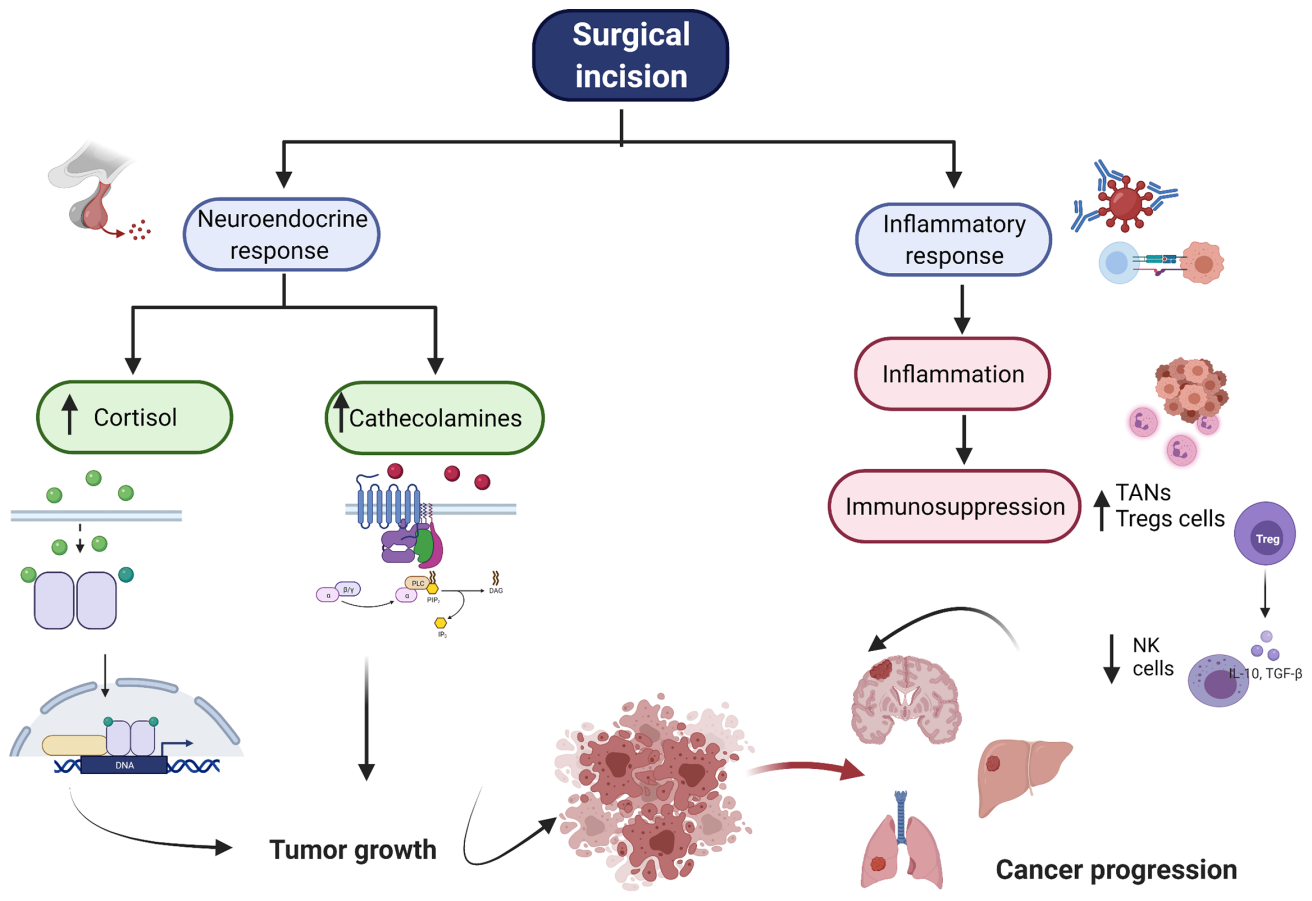
Overview of Surgical Stress Response. The figure represents the neuroendorine and the inflammatory response associated with surgery. After surgical incision, there is an increase of cortisol and cathecholamines. Additionally, there is a profound inflammatory response folllowed by immunosupression. All these together enables cancer cells to growth, proliferate and produce distant metastasis. NK, natural killer cell, TAN, tumor associated neutrophiles, Tregs, regulartoy T cells.

Cancer metastasis is the major cause of morbidity and mortality, and in fact it accounts for 90% of deaths in cancer patients (5). In order to successfully colonize a distant site CTCs must complete a sequence of events before they become clinically detectable metastasis. The development of metastasis therefore requires; 1) escape of tumor cells from primary tumor, 2) intravasation, 3) circulation in the blood stream, 4) extravasation through endothelial cells into the surrounding tissue, and 5) survival and proliferation in the TME by induction of angiogenesis and immune escape (**Figure 1**) (6). Also, an essential step on the metastatic process is the epithelial-mesenchymal transition (EMT). EMT allows the transformation of epithelial cancer cells into mesenchymal cancer cells (7). This phenotypic transformation enables mesenchymal cells to migrate, invade and resist apoptosis as they colonize distant sites. Cumulative evidence indicates that surgery increases the shedding of tumor cells into the circulation (8) and activates the sympathetic nervous response which ultimately triggers inflammation followed by immunosuppression (**Figure 2**) (9).

The initial acute inflammatory stress response is mediated by neutrophils, macrophages and monocytes at the site of injury. These immune cells release a massive production of pro-inflammatory cytokine including interleukin -1β (IL-1β), interleukin-6 (IL-6), tumor necrosis factor α (TNF-α) and neutrophils extracellular traps (NETs). All these cytokines shift CD4+ helper cells to a th1 profile (10). The Th1 profile, generally accepted as anti-tumoral, is characterized by the secretion of interferon gamma (INF)-γ and IL-2 with regulation of the cell mediated immunity (11). It is important to point out that the inflammatory response is directly proportional to the degree of surgical trauma. Human studies assessing the effect of minimally invasive versus open surgery have shown significant differences between the two interventions when reporting the function of immune cells and cytokine profile(12); “Inflammatory Response After Laparoscopic Versus Open Resection of Colorectal Liver Metastases Data From the Oslo-CoMet Trial: Erratum,” (13). For instance, laparotomy triggers higher concentrations of IL-6 than laparoscopic cancer surgery(“Inflammatory Response After Laparoscopic Versus Open Resection of Colorectal Liver Metastases Data From the Oslo-CoMet Trial: Erratum,” (13).

The surgical inflammatory response is followed by a compensatory anti-inflammatory response; however it can also lead to dysregulation of the cell mediated immunity with subsequent immunosuppression (14). IL-6 induces the release of prostaglandin E_2_ (PGE_2_) from macrophages (15). PGE2 is a lipid mediator that exerts its activity *via* PGE_2_ receptors (EP1-4). EP2 and EP4 are both G_s-_couple receptors that signal through the adenylate cyclase-dependent cAMP/PKA/CREB pathways (16). The effects of PGE_2_ includes the inhibition of neutrophil, natural killer (NK) and T-cell mitogenesis (17). Furthermore, protanglandin regulates lymphatic vessles dilatation and therefore could enables cancer mestatasis (18). Additionally, PGE_2_ inhibits the production of IL-1β, IL-6 and TNF-α and stimulates the release of IL-10, IL-1Ra (19). This cytokine imbalance results in a shift toward th2 profile (pro-tumoral), which favors tumor growth by inhibiting cell-mediated immunity (20).

The stress response to surgery is also characterized by the secretion of cortisol and catecholamines (21). Cortisol can diffuse the cellular membrane to bind the glucocorticoid receptor intracellularly. This complex, then translocates into the nucleus where it interacts with glucocorticoid-responsive elements (DNA sequence) and different transcription factors such as NF-kB to inhibit or promote the production of inflammatory cytokines (22). For instance, cortisol has shown a dual role in oral squamous cell carcinoma. At physiological stress levels (i.e., 10 nM) cortisol promoted the expression of IL-6 while higher pharmacological concentration (i.e., 1000 nM) produced the opposite findings (23). The sympathetic nervous system directly modulates cancer cells *via* β-adrenoreceptors-mediated activation of protein kinase A (PKA) (24). β-adrenoreceptors have been found in breast, prostate, lung, esophageal and liver cancer cells among others (25–29). The activation of β-adrenergic signaling by epinephrine or norepinephrine triggers an increase on cyclic adenosine monophosphate (cAMP) which directly modulate cancer cell growth, proliferation, invasiveness, angiogenesis and metastasis (24). One characteristic of cancer cells is the formation of invadopodia (actin-rick protrusions) which are formed to degrade and facilitate migration through the extracellular matrix (30). β-adrenoreceptors activation can promote an increase of invadopodia which correlates with increased tumor invasion in *in vivo* breast cancer models. Importantly, such effect is reversed by β-blockers (31).

### Surgery Induces Angiogenesis

A critical step in the metastatic process is the development of new blood vessels (angiogenesis). The vascular endothelial growth factor (VEGF), an extensively studied molecules in angiogenesis, is considered a maker of poor prognosis for some cancers (32, 33). VEGF as well as its receptors (VGFR1 and VGFR2) have been found in cancer cells (34). The activation of VEFGR initiates MAPK signaling pathway with phosphorylation of ERK and ultimately promotion of cell proliferation (35). VEGF has been reported to be higher in cancer patients compared to control groups even before surgery (36–39). It has been theorized that high perioperative levels of VEGF might explain why cancer surgery might facilitate the growth of residual metastases disease early after surgery.

### Key Effectors Cells of the Immune Response in Cancer Surgery

Neutrophils are the first line to respond to surgical trauma and defend against invading microorganisms. However, neutrophils have been shown to play a dual role since besides protecting from infection, neutrophils can also lead to cancer progression and tumor dissemination (40). Tumor associated neutrophils (TANs) are associated with poor overall survival in many types of cancers (41–43). Neutrophils can serve as chemotactic factor to attract cancer cells by releasing neutrophil extracellular trap (NETs)(L. 44). Surgery triggers the formation of NETs which can promote formation of metastasis. The inhibition of NETs after surgery powerfully counteract their pro-metastatic effects (45).

Natural killer cells are one of the main effector cells against cancer (46). Upon target cell recognition, NK cells mediate target cell lysis by two different mechanisms. First, the release of cytotoxic granules containing granzyme and perforins, and the induction of Fas ligand and TNF-related apoptosis ligand (TRAIL) (47). Second, activated NK cell secrete several cytokines such as INF- γ, TNF-α and chemokines (i.e., CCL3, CCL 4 and CCL5). Accumulated evidence suggests that NK cell cytotoxicity is decreased immediately after surgery secondary to surgical stress. This effect can last for several weeks (48). Additionally, the surgical stress impair the NK cells’ capacity to secrete INF-γ and therefore decreases the activation of the cellular immunity and subsequently antitumor immune response (49). The extent of the surgical insult impacts the function of these cells. For instance, laparoscopically assisted surgery resulted in better preservation of NK cell function compared to open procedures in patients with colon cancer (50).

Lymphocytes are an essential component for maintaining tolerance and preventing excessive inflammation. Postoperative lymphopenia or a high neutrophil-to-lymphocyte ratio (NLR) are independent biomarkers of cancer recurrence (51–53). NLR appears to be an appealing biomarker in cancer prognosis since its widely available, easily measured and inexpensive. A recent meta-analysis by Cupp et al. suggested an association between high NLR and poor cancer outcomes (54). For instance, Forget *et al*. demonstrated that preoperative high NLR in patients with breast, lung and renal cancer undergoing tumor resection was associated with higher risk or relapse and/or higher mortality (55). Similar findings in term of RFS and OS were found by Choi *et al.* in a cohort of non-small cell lung cancer (NSCLC) patients, however the correlation was only observed in patient with Stage I NSCLC (56). Among lymphocytes, regulatory T cells (Tregs) are also regulators of the anti-tumor immunity (57). Ghiringhelli et al. reported a high Tregs cell levels that correlated with a low number of NK cells that were also dysfunctional in gastrointestinal stromal tumor-bearing patients (58). Peripheral and tumor infiltrating Tregs levels are higher in patients with breast and pancreas cancer compared to healthy subjects. High levels of circulating tumor infiltrating Tregs have been associated with accelerated progression and poor prognosis of those cancers (59). While in the context of low levels of Tregs can predict the presence of postoperative complications, the impact of different peripheral concentrations of these cells after oncological procedures is less understood (60).

In summary, the perioperative period is critical for several steps leading to cancer metastasis. It has been indicated that anesthetics could also influence mechanisms such as NETs formation, EMT and angiogenesis. In the following section, we will summarize the preclinical and clinical evidence regarding the effects of the different types of anesthesia techniques on long-term cancer outcomes.

## Inhalational Agents and Intravenous Anesthetics for Cancer Surgery

### Preclinical Evidence

#### Volatile Anesthetics

Volatile anesthetics are commonly used during oncological surgery. There has been increasing interest in investigating the role of volatile anesthetics on cancer recurrence and metastasis. Preclinical data suggest that volatile agents promote the progression of cancer by direct and indirect mechanisms. Firstly, volatile anesthetic can directly modify (by either promoting or inhibiting) intracellular signals involved in key aspect of the cancer cell behavior such as proliferation, migration, invasion and sensitivity to chemotherapeutic agents. For instance, isoflurane (1.2%) increased the proliferation and migration while decreasing apoptosis in glioblastoma stem cells by regulating the expression of hypoxia-inducible factor (HIF) (61, 62). In non-small cell lung cancer, isoflurane at 1%, 2% and 3% promoted proliferation, invasion and invasiveness *via* Akt-mTOR signaling (63). In a colorectal cancer cell line, desflurane (10.3%) induces EMT and metastasis through dysregulation of miR-34/LOXL3 axis a well-known tumor suppressor (64). Sevoflurane (2% for six hours), *in vitro*, increases survival of breast cancer cells *via* modulation of intracellular Ca^2+^ homeostasis (65). Secondly, volatile anesthetics could facilitate cancer progression by inducing immunosuppression. For example, sevoflurane and desflurane attenuated NK cell cytotoxicity *in vitro* by inhibiting the expression of the adhesion molecule leucocyte- function n associated antigen (LFA-1) (66). In addition, isoflurane reduced the ability of NK cells to respond to INF-γ stimulation. A phenomenon that lasted for 11 days (67). Importantly, sevoflurane, isoflurane and enflurane at 1.5 and 2.5 MAC reduced the release of TNF-α and IL-1β in human peripheral blood mononuclear cells (68).

Contrary to this previously cited evidence, a number of preclinical studies indicate that volatile anesthetics might have an anti-tumoral effect. For instance, concentration of sevoflurane from 1.7% to 5.1% significantly inhibits invasion and migration of lung carcinoma cells by decreasing the phosphorylation of p-38 MAPK, reducing HIF-1α activation and downregulating matrix metallopeptidases (MMP) 2 and MMP-9 (69–71). In colon cancer, sevoflurane induced p53-dependent apoptosis while suppressing cell migration and invasion by regulating the ERK/MM-9 pathway (via miR-203) (72, 73). Lastly, sevoflurane at clinical (2.5%) and toxic concentrations (5% and 10%) inhibited viability, migration and invasion of osteosarcoma cells by inactivating PI3K/ATK pathway (74).

In summary, volatile anesthetics regulate important functions in cancer cells. Their inconsistent (pro and anti-tumoral) effects cancer cells and those of the TME could be explained by differences in experimental conditions such as, type of cell line, incubation time (ranged between 30 mins and 6 hours), type and concentration of volatile anesthetics (ranged between 0.5%-10%). For instance, some studies treated cancer cells with very high concentrations that are not usually employed in clinical practice and perhaps the “anti-tumoral” effect is most likely related to toxic concentrations of volatile anesthetics.

### Propofol

Propofol based total intravenous anesthesia has gained attention in recent years. Most preclinical studies suggest that propofol inhibits tumor cell viability, proliferation, migration and invasion by regulating different signaling pathways. It inhibits proliferation, migration and invasion in colon cancer cells by upregulating miR-124-3p and downregulating AKT3 (75). Also in colon cancer, propofol decreases cell invasion *via* ERK1/2-depenedent downregulation of MMP-2 and -9 (76). In lung cancer cells, propofol promotes apoptosis also *via* ERK1/2 *via* activation and upregulation of p53 (77), and decreases metastatic cell behaviors by inhibiting HIF-1α (78) and MMPs-2,-7 and -9 (79). Similarly, it inhibits migration of breast cancer cells by inhibiting MMP expression *via* NF-κB pathway (80). In glioma cells, propofol reduced migration and invasion by blocking PI3K/AKT pathways *via* mi-R-206/ROCK1 axis (81). Moreover, propofol reduced oxidative stress and growth in glioma cells by suppressing the Ca^2+^-permeable α-amino-3-hydroxyl-5methylisoxazole-4-propionic acid (AMPA)receptor and divalent transporter 1(DMT1) (82).

The anti-tumoral effect of propofol in cancer progression also entails indirect mechanisms such as the potentiation of NK cell cytotoxicity and reduction of inflammatory response. For instance, in colon cancer cells, propofol increased expression of activated receptor p30 and p44 in NK cells, which promoted NK cell activation and proliferation (83). Additionally, in esophageal squamous cell carcinoma cells, propofol enhanced the expression of cytotoxic effector molecules like granzyme B and IFN-γ suggesting that NK cytotoxicity was increased (84). In terms of cytokine profile, propofol decreases pro-inflammatory cytokines such as IL-1β, IL-6 and TNF-α (85) and inhibits PGE2 and COX activity (86). Moreover, propofol decreased NETs formation (through inhibition of p-ERK) without affecting neutrophil killing capacity (87, 88).

Altogether, propofol preferentially promotes anti-metastatic mechanism in cancer cells and those of the TME.

### Intravenous Ketamine, Dexmedetomidine and Lidocaine

Ketamine is routinely used during cancer surgery to provide analgesia and reduce the use of volatile anesthetics and opioids. Increasing number of studies suggest that ketamine can modify proliferation and survival of cancer cells (89). For example, ketamine decreased intracellular Ca^2+^, expression of HIF-1α, p-AKT, p-ERK with subsequent reduction of VEGF expression and cell migration in colorectal cancer cells. Notably, all these changes were associated with NMDA receptor inhibition since D-serine (NMDA activator) reversed the anti-tumoral effect of ketamine (90). Additionally, ketamine promotes apoptosis and inhibits cell growth proliferation in lung adenocarcinoma; throughout CD69 expression (91), hepatic cell carcinoma; throughout Bax-mitochondria-caspase protease pathway (92); pancreatic carcinoma *via* NMDA receptor type R2a (93) and ovarian cancer through the inhibition of long-non-coding RNAs PVT1 expression (89).

Dexmedetomidine has also gained interest due to its sedative and analgesic effects. In esophageal carcinoma, dexmedetomidine inhibits tumor growth and metastasis *via* upregulation of miR-143-3p and reduction of levels of epidermal growth factor receptor 8 (94). Additionally dexmedetomidine enhances immune surveillance by inhibiting the p38 MAPK/NF-κB signaling pathway; however, some authors have indicated that dexmedetomidine can stimulate proliferation of cancer cells (95, 96). For instance, dexmedetomidine induced secretion of IL-6 and promoted progression *via* STAT 3 activation in hepatocellular carcinoma (97). Similarly, it promoted tumor proliferation and migration *via* adrenergic signaling and upregulation of Bcl-2 and Bcl-xL (anti-apoptotic proteins) in neuroglioma and lung carcinomas (98). In a rodent model of breast, lung and cancer colon, dexmedetomidine promoted tumor growth and metastasis (99).

Lidocaine is an amide local anesthetic that has gained popularity because of its anti-ileus effects and suggested beneficial properties in recovery after surgery. Lidocaine suppress tumor cells directly by modifying cancer cells signaling. For instance, lidocaine inhibited metastasis and proliferation of lung cancer cells by up-regulating miR-539 with subsequent blocking of EGFR signaling (100). Furthermore, lidocaine suppressed hepatocellular cell growth and induced apoptosis (via activation of caspase- 3 and regulation of Bax/Bcl-2 proteins through the MAPK pathway)( 101). Likewise, lidocaine inhibited cervical cancer cell growth and induced apoptosis by modulating lrnRNA-MEG3/miR-421/BTG1 pathway (102).

Lidocaine has shown potent anti-inflammatory properties by decreasing both; pro-inflammatory cytokines (IL-1β, IL-6 and TNF-α) and intercellular adhesion molecules (I-CAM) expression (103, 104). Human studies have also confirmed this finding in a randomized controlled trial (RCT) where intravenous lidocaine was associated with significantly less production of IL-1ra, IL-6 with preservation of the lymphocyte proliferation (105). Lidocaine has also stimulated the function of NK cells of patients undergoing cancer surgery (106). Recently, a RCT looking at the effect of intravenous lidocaine infusion in breast cancer patients demonstrated a decrease in postoperative expression of NETosis (which is associated with disease progression) and MMP3 (107). Lastly, lidocaine has shown anti-angiogenic effects. It decreased, in a dose dependent manner (1-10µg/ml) the expression of VEGF-A. The inhibitory effects were the result of inhibition of VEGFR-2 phosphorylation (108).

Taken together, experimental evidence suggests that volatile anesthetics might promote tumor progression by directly modifying intracellular signals involved in key aspects of cancer cell behavior such as proliferation, migration and invasion. Additionally, volatile anesthetics might promote immunosuppression. In contrast, propofol has shown anti-inflammatory properties and potentiation of the immune response. Data for ketamine and dexmedetomidine is inconsistent with some studies showing promotion of tumor progression while other showing opposite findings. On the other hand, lidocaine has shown promising results.

## Clinical Evidence

Retrospective studies (**Table 1**) indicate that cancer survival and recurrence could be affected by the anesthetic technique. The most recent systematic review and meta-analysis by Chang et al. included 19 retrospective observational studies of patients undergoing surgery for various types of cancer surgery. (130) Pool analysis of OS included 17 studies with 23,489 patients (110, 113–119, 121–124, 126, 128, 131, 132). The study showed that propofol-based TIVA in cancer surgery was associated with better OS compared to volatile agents (HR= 0.79, 95% CI, 0.66-0.94, p= 0.08). Interestingly the results of the subgroup analysis by volatile anesthetics showed that this benefit was statistically significant only when TIVA was compared to desflurane (HR= 0.54, 95% CI, 0.36-0.80, p= 0.03), but not compared to sevoflurane (HR=0.92, 95% CI, 0.74-1.14, p=.436) or other volatile agents (HR=0.83, 96% CI, 0.64-1.07, p=0.156). In terms of RFS, the study pooled the results of 10 studies with 8,980 patients (110, 113, 114, 116, 117, 123, 124, 126, 127, 132). The analysis indicated no benefits in survival when using TIVA compared to volatile agents (HR=0.92, 95% CI, 0.74-1.14, p=0.439).

**Table 1:** Retrospective trials comparing the effect of TIVA versus volatile anesthesia on long-term cancer outcomes.

Type of Cancer	Author	Overall Survival	Recurrence- Free Survival
Gastrointestinal	(109)	No difference	No difference
Hepatocellular	(110)	No studied	Increased with TIVA
Glioblastoma	(111)	No difference	No difference
Breast	(112)	No difference	No difference
Glioma	(113)	No difference	No difference
Breast	(114)	No difference	No difference
Gastric	(115)	No difference	No difference
Cholangiocarcinoma	(116)	Increased with TIVA	No difference
Hepatocellular	(117)	Increased with TIVA	Increased with TIVA
Breast	(118)	No difference	No difference
Breast, Liver, Lung and Gastrointestinal	(119)	No difference	No difference
Appendiceal	(120)	No difference	No difference
Gastric	(121)	Increased with TIVA	No studied
Colon	(122)	Increased with TIVA	No studied
Lung	(123)	No difference	No difference
Breast	(124)	No difference	No difference
Glioblastoma	(125)	No difference	No difference
Esophageal	(126)	No difference	No difference
Breast	(127)	No difference	No difference
Breast, Sarcoma Gastrointestinal and Urologic	(128)	Increased with TIVA	No studied
Ovarian	(129)	No studied	Increased with volatile anesthetic

Interestingly the benefits in OS in Chang’s work were seen in patients with gastrointestinal malignancies, which is the same type of cancers included in another study done by Yap et al (133). Importantly, this group of investigators found that the use of propofol–based TIVA not only improved OS (HR= 0.76, 95% CI, 0.63-0.92, p <0.01) but also improved RFS (HR=0.78, 95% CI, 0.65-0.94, p<0.01). There are some important study limitations that need to be highlighted when analyzing the available meta-analyses. For example, Wigmore et al. study acknowledged the difference in the baseline characteristics between groups, with more ASA III/IV patients, more complex surgeries and larger metastatic burden in the volatile anesthetics group. Nevertheless after propensity matching to correct potential confunders, the study groups were similar (128). Lai’s study presented the same limitation for hepatocellular carcinoma surgery. In that study, the desflurane group had significantly more patients with worse preoperative functional capacity, higher scores of liver disease and tumor grade staging compared to the propofol-based group. Patients in the desflurane group were also more likely to have larger tumors and receive blood transfusions which are all independent factor associated with decreased survival (117).

It is important to point out that the systematic review conducted by Chang et al. included studies published until March 2020 and unfortunately did not include the largest retrospective study done by Makito et al. (which was published later in the same year) (109). In that retrospective study the author investigated the effect of TIVA and volatile agents on long-term oncological outcomes among 196,303 patients with gastrointestinal malignancies and found that OS (HR= 1.02, 95% CI, 0.98-1.07, p= 0.28) and RFS (HR=0.99, 95% CI, 0.96-1.03, P= 0.59) were similar between propofol-based TIVA and volatile anesthetic groups. Similar to Makito’s work, other multiple retrospective studies showed no difference between TIVA and volatile in terms of OS and RFS in patients with breast cancer (114, 118, 124, 131). The lack of benefit from propofol-based TIVA has also been described for lung (123, 134) and brain cancer surgeries (111, 113, 125). Subsequent substudies from RCTs in lung and breast cancer indicated the same results (134–136). However, it is important to point out that these RCTs did not have OS and RFS as primary outcome.

Since retrospective studies have significant limitations, RCTs are necessary to determine whether the use of propofol-based anesthesia modifies cancer outcomes in patients undergoing surgery for solid tumors (**Table 2**). The VAPOR-C trial (NCT04074460) has a 2x2 factorial design and will investigate the impact of TIVA vs. inhalational agents and lidocaine vs. placebo on DFS after lung and colorectal cancer surgery with curative intent (stage 1-3) (138). The cancer and anesthesia study (NCT01975064) is also investigating the effect of propofol-based TIVA versus volatile anesthesia in breast and colon cancer patients. Preliminary data for 1-year survival is already available and unsurprisingly no benefit was observed in the propofol-based TIVA group (137). The results from long-term survival (5 years) are expected to be available for 2022-2023. The GA-CARES trial (NCT03034096) will randomize 2,000 patients to assess all-cause mortality and RFS in patient undergoing lung, bladder, esophagus, pancreas, liver, gastric and biliary duct cancer surgery with propofol-based anesthesia or volatile anesthetics.

**Table 2:** Randomized control trials comparing the effect of TIVA versus volatile anesthesia on long-term cancer outcomes.

Type of Cancer	Author	Overall Survival	Recurrence- Free Survival
Breast	(137)	*No difference	No published yet
Breast	(135)	No difference	No difference
Breast	(136)	No difference	No difference
Lung	(134)	No difference	No difference

*Preliminary data from 1 year OS.

The effect of intravenous lidocaine on cancer outcomes was recently investigated in pancreatic surgery. A retrospective study of more than 2,239 patients assessed the effect of intraoperative lidocaine (bolus injection of 1.5 mg/kg followed by continues infusion 2mg/kg/hour) and suggested that intravenous lidocaine was associated with prolonged OS (HR=0.616, 95% CI, 0.290-0.783, p=0.013), but not DFS (HR=0.913, 95% CI, 0.821-1.612, p=0.011) (139).

In conclusion, the current evidence is weak to indicate that propofol-based general anesthesia provides any oncological benefit to patients with cancer requiring surgery.

## Regional Anesthesia Compared to General Anesthesia for Cancer Surgery

Regional anesthesia (RA) techniques including peripheral nerve blocks and neuraxial anesthesia were associated with a reduction in cancer recurrence in preclinical and observational studies. it was originally theorized that RA could improve oncological outcomes after cancer surgery since RA decreases the neuro-endocrine response to surgical trauma, opioid consumption and the use of volatile anesthetics (140–142). Additionally, RA preserves the function of the immune system and has a direct inhibitory effect on cancer cells (143, 144).

## Clinical Evidence

Thus far, the evidence regarding the potential benefits of RA in long-term outcomes originates from preclinical, retrospective, *post hoc* analysis of RCT and few RCTs (**Tables 3**, **4**). The most recent RCT enrolled 400 patients to investigate the effect of combined epidural-general or general anesthesia alone in patients undergoing video-assisted thoracoscopic lung cancer resection. The primary outcome was RFS. Secondary outcomes were OS and cancer-specific survival. The median follow-up was after 32 months. Results indicated that epidural-anesthesia for major lung surgery did not improved RFS (HR=0.90, CI 95% 0.60-1.35, p=0.068), cancer–specific survival (HR=1.08, CI 95% 0.61-1.91, p=0.802) or OS (HR=1.12, CI 95% 0.6401.96, p=0.697) compared to general anesthesia alone (172),

**Table 3:** Retrospective trials assessing the effect of regional anesthesia on long-term cancer outcomes.

Type of Cancer	Author	Intervention	Overall Survival	Cancer Recurrence
**Colon**	(145)	Epidural	No benefit from RA	No benefit from RA
**Colon**	(146)	Epidural	No benefit from RA	Benefit from RA
**Colon**	(147)	Epidural	Benefit from RA	No reported
**Colon**	(148)	Epidural	Benefit from RA	No reported
**Colon**	(149)	Epidural	Benefit from RA	No benefit from RA
**Colon**	(150)	Epidural	No benefit	No benefit
**Colorectal**	(151)	Epidural	Benefit from RA	No reported
**Colon**	(152)	Epidural	No reported	No benefit
**Breast**	(153)	Loco-regional anesthesia	No benefit from RA	No benefit from RA
**Breast**	(154)	Paravertebral block	No benefit from RA	No benefit from RA
**Breast**	(155)	Paravertebral block	No benefit from RA	No benefit from RA
**Breast**	(156)	Paravertebral block	No reported	No benefit from RA
**Breast**	(157)	Epidural Paravertebral block	No reported	No benefit from RA
**Breast**	(158)	Paravertebral block	No reported	Benefit from RA
**Prostate**	(159)	Spinal	No reported	No benefit from RA
**Prostate**	(160)	Epidural	No benefit from RA	No benefit from RA
**Prostate**	(161)	Spinal	No reported	No benefit from RA
**Prostate**	(162)	Spinal	No benefit from RA	No benefit from RA
**Prostate**	(163)	Spinal	No reported	No benefit from RA
**Prostate**	(164)	Epidural	No benefit from RA	No benefit from RA
**Prostate**	(165)	Epidural	No reported	No benefit RA
**Prostate**	(166)	Epidural	No benefit from RA	Benefit from RA
**Prostate**	(167)	Epidural	No reported	Benefit from RA
**Ovarian**	(129)	Epidural	No reported	Benefit from RA
**Ovarian**	(168)	Epidural	No benefit from RA	No benefit from RA
**Ovarian**	(169)	Epidural	No benefit from RA	No benefit from RA
**Ovarian**	(170)	Epidural	Benefit from RA	No reported
**Ovarian**	(171)	Epidural	No reported	Benefit from RA

**Table 4:** Randomized control trials assessing the effect of regional anesthesia on long-term cancer outcomes.

Type of Cancer	Author	Intervention	Overall Survival	Cancer Recurrence
**Lung**	(172)	Epidural	No benefit from RA	No benefit from RA
**Thoracic and Abdominal**	(173)	Epidural	No benefit from RA	No benefit from RA
**Breast**	(135)	Paravertebral block	No reported	No benefit from RA
**Breast**	(174)	Paravertebral block	No benefit from RA	No benefit from RA
**Breast**	(175)	Paravertebral block	No reported	No benefit from RA
**Colon**	(176)	Epidural	No benefit from RA	No benefit from RA
**Colon**	.(177)	Epidural	No benefit from RA	No benefit from RA
**Colon**	(178)	Epidural	Benefit with RA	No reported
**Prostate**	(179)	Epidural	No reported	No benefit from RA

The effect of combined epidural-general was also investigated in a large RCT including patients (n= 1,712) undergoing major non-cardiac thoracic or abdominal surgery. The median follow-up time was after 5 years. Again, mortality (HR=1.07, CI 95% 0.92- 1.24, p=0.408), cancer-specific survival (HR=1.09, CI 95% 0.93-1.28, p=0.290) and RFS (HR=0.97, CI 95% 0.84-1.12, p=0.692) was similar between combined epidural-general anesthesia and general anesthesia group. (173) In the setting of breast cancer surgery, two RCTs also failed to demonstrate any benefits from paravertebral blocks in terms of cancer outcomes in patients undergoing breast cancer surgery (135, 174). Other RCTs looking at the effect of RA on colon and prostate cancer surgery also failed to demonstrate any benefits in cancer outcomes (177, 179).

There are multiple RCTs in progress to determine the effects of RA compared to general anesthesia on cancer progression. The study NCT03597087 will assess RFS and PFS in patients undergoing transurethral resection of bladder tumors under spinal anesthesia. NCT03245346 will investigate the effect of epidurals on OS and RFS in patients undergoing pancreatic cancer surgery. This trial will also assess the inflammatory neuro-endocrine response by measuring norepinephrine, epinephrine, cortisol and IL-6, IL-8 levels and by measuring the neutrophil-lymphocyte ratio. Lastly, NCT02786329 will investigate the effect of epidural anesthesia in patients undergoing lung cancer resection.

In conclusion, a growing body of evidence from RCTs consistently demonstrates that cancer-specific mortality and cancer recurrence are not improved by the use of regional anesthesia during oncologic surgery.

## Conclusion

Cancer surgery remains the standard of care for patients with solid tumors. Despite curative intent, 90% of cancer mortality is secondary to cancer metastasis. Preclinical data suggest that the perioperative stress response to surgical trauma creates a window of opportunity for accelerated tumor growth and metastasis. This effect seems to be secondary to changes in signaling pathways in both-TME and immune response. Total intravenous anesthesia and regional anesthesia have been proposed as strategies to counteract the inflammatory response and the associated immunosuppression associated with cancer surgery. Unfortunately, the majority of the data looking at the relationship of these techniques and cancer outcomes originates from retrospective studies. Whether volatile anesthetics have a deleterious effect of cancer recurrence and survival remains a controversial issue. RCTs are in progress and will explore a causal relationship between volatile anesthetic and cancer outcomes. As far for regional anesthesia, RTCs have consistently shown lack of benefit of this technique in regards to cancer survival and recurrence.

## Author Contributions

MR and JC: discussed ideas and prepared the manuscript. MR: prepared figures and tables. JC: improved manuscript and edited. All authors contributed to the article and approved the submitted version.

## Conflict of Interest

The authors declare that the research was conducted in the absence of any commercial or financial relationships that could be construed as a potential conflict of interest.

## Publisher’s Note

All claims expressed in this article are solely those of the authors and do not necessarily represent those of their affiliated organizations, or those of the publisher, the editors and the reviewers. Any product that may be evaluated in this article, or claim that may be made by its manufacturer, is not guaranteed or endorsed by the publisher.
